# Metabolism: A Novel Shared Link between Diabetes Mellitus and Alzheimer's Disease

**DOI:** 10.1155/2020/4981814

**Published:** 2020-01-29

**Authors:** Yanan Sun, Cao Ma, Hui Sun, Huan Wang, Wei Peng, Zibo Zhou, Hongwei Wang, Chenchen Pi, Yingai Shi, Xu He

**Affiliations:** ^1^Key Laboratory of Pathobiology, Ministry of Education, College of Basic Medical Sciences, Jilin University, Changchun 130021, China; ^2^Department of Pathology, Zhongda Hospital, School of Medicine, Southeast University, Nanjing 210009, China; ^3^Department of Clinical Medicine, Jilin University, Changchun 130021, China; ^4^The First Hospital, Jilin University, Changchun, Jilin 130021, China

## Abstract

As a chronic metabolic disease, diabetes mellitus (DM) is broadly characterized by elevated levels of blood glucose. Novel epidemiological studies demonstrate that some diabetic patients have an increased risk of developing dementia compared with healthy individuals. Alzheimer's disease (AD) is the most frequent cause of dementia and leads to major progressive deficits in memory and cognitive function. Multiple studies have identified an increased risk for AD in some diabetic populations, but it is still unclear which diabetic patients will develop dementia and which biological characteristics can predict cognitive decline. Although few mechanistic metabolic studies have shown clear pathophysiological links between DM and AD, there are several plausible ways this may occur. Since AD has many characteristics in common with impaired insulin signaling pathways, AD can be regarded as a metabolic disease. We conclude from the published literature that the body's diabetic status under certain circumstances such as metabolic abnormalities can increase the incidence of AD by affecting glucose transport to the brain and reducing glucose metabolism. Furthermore, due to its plentiful lipid content and high energy requirement, the brain's metabolism places great demands on mitochondria. Thus, the brain may be more susceptible to oxidative damage than the rest of the body. Emerging evidence suggests that both oxidative stress and mitochondrial dysfunction are related to amyloid-*β* (A*β*) pathology. Protein changes in the unfolded protein response or endoplasmic reticulum stress can regulate A*β* production and are closely associated with tau protein pathology. Altogether, metabolic disorders including glucose/lipid metabolism, oxidative stress, mitochondrial dysfunction, and protein changes caused by DM are associated with an impaired insulin signal pathway. These metabolic factors could increase the prevalence of AD in diabetic patients via the promotion of A*β* pathology.

## 1. Introduction

As a chronic metabolic disease, diabetes mellitus (DM) is one of the most important public health challenges in the 21st century. DM is broadly characterized by elevated levels of blood glucose, mainly caused by insufficient insulin production or unresponsiveness of the body to insulin. According to the latest data from the International Diabetes Federation (IDF, https://www.diabetesatlas.org/), there are 425 million people with diabetes in the world, and one in 11 adults worldwide is diagnosed as having DM. By 2045, the number of people with DM is expected to increase to 629 million. Recent epidemiological studies have shown that some diabetic patients are more susceptible to dementia than healthy individuals [1–4]. In addition, mounting statistical and biological data support a correlation between DM and dementia [5–9], which may share common cellular and molecular mechanisms.

Based on a recent report from the World Health Organization (WHO), approximately 50 million people worldwide have dementia. Although there are over 100 types of dementia, the most well-known form is Alzheimer's disease (AD), which accounts for 50%-75% of all cases. AD can damage cells and nerves interrupting the transmitters that convey information in the brain, particularly those responsible for memory storage. Generally, gradual memory loss is the first symptom of AD, but other signs include difficulty in finding the right word, correctly identifying other people, and solving problems [10–12]. In addition, the majority of AD cases frequently suffer from multiple complications (e.g., DM, other neurodegenerative disorders, cardiovascular diseases, and renal diseases). These comorbidities can enhance the complexity that underlies AD pathogenesis [13–15].

Until recently, studies have suggested that these complications may be associated with AD pathogenesis and memory loss [16–18]. Among the various complications, DM has been shown to be the most influential in the development of AD [13, 14]. Epidemiological studies of AD in diabetic patients from the past decade are shown in Table 1. From these data, we conclude that high AD prevalence in some diabetic populations may be related to insulin resistance and metabolic abnormalities. However, not all individuals with DM develop dementia, and not all dementia cases have DM. DM and AD are two independent metabolic diseases, and both of them are associated with disturbed energy metabolism in the body. Therefore, we speculate that the increased risk of AD in diabetic patients may be partially attributed to metabolic abnormalities. As one of the most basic features of an organism, energy metabolism is a reactive steady-state system that satisfies the energy demands of tissues and organs [19]. Improved understanding of the metabolic associations between DM and AD might provide novel insight into the onset and relationship between both diseases and may at least partly explain the causality.

## 2. Impaired Insulin Signaling Pathway: The Pathogenesis of AD

**Figure 1:**
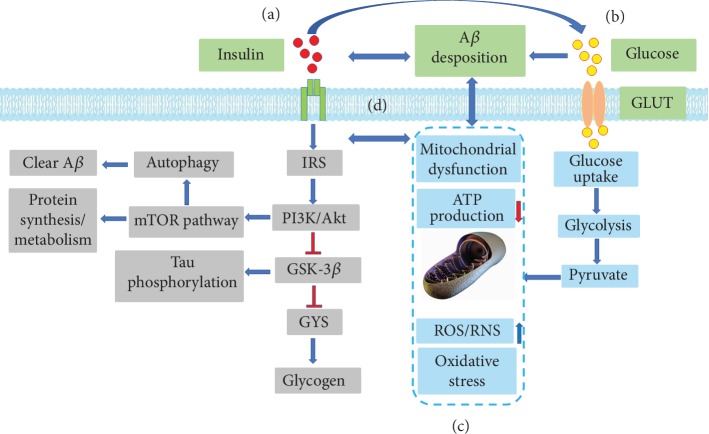
Overview of the insulin signaling pathway and relevant mechanisms implicated in AD. (a) Tyrosine phosphorylation of insulin receptor substrates (IRS) allows the association of IRSs with the regulatory subunit of phosphoinositide 3-kinase (PI3K). The PI3K/AKT signaling pathway deactivates glycogen synthase kinase 3 (GSK-3), leading to the activation of glycogen synthase (GYS) and thus glycogen synthesis. In addition, the PI3K/AKT signaling pathway can affect protein synthesis/metabolism and clearance of A*β* by activating the mTOR pathway. (b) The insulin/insulin signaling pathway can promote glucose transportation by regulating GLUT, and high levels of glucose can lead to A*β* deposition. (c) Disordered glucose metabolism leads to ROS/RNS formation and decreased ATP production, which is the main manifestation of oxidative stress. Mitochondrial dysfunction can lead to impaired cellular energy production and reduction in insulin secretion and sensitivity. (d) Impaired insulin signaling pathway, A*β* deposition, and mitochondrial dysfunction promote each other to form a vicious circle.

Insulin, a peptide hormone produced by the beta cells of the pancreatic islets, is the main anabolic hormone of the body and can regulate the metabolism of carbohydrates, lipids, and proteins. The first step in triggering the insulin signaling pathway is insulin recognizing and binding to a transmembrane tyrosine kinase receptor. This receptor (insulin receptor (IR)) results in the tyrosine phosphorylation of insulin receptor substrates (IRS) by the insulin receptor tyrosine kinase (INSR), thereby blocking downstream signaling pathways such as the phosphatidylinositol 3-kinase/protein kinase B (PI3K/AKT) pathway. The PI3K/AKT pathway further regulates a number of downstream signaling pathways important for protein synthesis and amyloid-*β* (A*β*) clearance (i.e., through mammalian target of rapamycin (mTOR) signaling) [20]. This pathway also affects the activity of glycogen synthase kinase-3 (GSK-3*β*), a major tau phosphorylating kinase and crucial glycogen synthase, which can modulate tau expression [21–23]. In addition, an impaired insulin signaling pathway, A*β* deposition, and mitochondrial dysfunction can interact reciprocally to form a vicious circle. This may explain why diabetic patients are susceptible to AD (Figure 1).

Similar to most organs in the body, the brain is an insulin-sensitive organ, where insulin signaling regulates energy metabolism, cell survival, and cellular homeostasis. Due to its neuroprotective function [24, 25], insulin is beneficial for neuronal growth and survival [26–28]. Over the past few years, accumulating data indicate that insulin regulates the concentration of several neurotransmitters that have essential roles in memory formation, such as acetylcholine, norepinephrine, and epinephrine [29]. Insulin also supports neuronal plasticity and cholinergic functions, which are required for learning, memory, and myelin maintenance. Moreover, damaged insulin signaling in the brain can greatly affect cognitive impairment and neurodegeneration, particularly mild cognitive impairment and AD [30]. Collectively, these observations suggest that insulin is a protective factor for brain function, and insulin signaling is an important pathway in the prevention of cognitive decline.

A*β* is the main component of senile plaques (SPs), which are one of the histopathological markers of AD, and has a vital influence on the progression of neurodegenerative diseases [31]. A previous work has found that pre-proinsulin mRNAs and insulin protein levels are significantly decreased by A*β*-stimulation in astrocytes [32]. Recent postmortem analyses also support the idea that insulin signaling is impaired in AD brains [33, 34]. Furthermore, deficient insulin signals have been observed in mammalian models of AD, in which A*β* is overexpressed or induced by an intracerebral injection [33, 35]. A*β* can also competitively inhibit insulin binding to the IR, which results in the loss of membrane insulin receptors. This is an important contributor to synaptic and dendritic spine damage [36], although there are other factors that contribute to synaptic dysfunction in AD such as oxidative stress, tau phosphorylation, and lipid peroxidation [37–39]. Taken together, these data suggest that A*β* pathology could contribute to insulin signaling destruction and that insulin signaling may be a potential target for the prevention and treatment of AD.

## 3. Glucose Metabolism Disorder Is a Potential Factor for AD in Diabetic Patients

Compared with energy demand, brain energy storage is limited, and reduced oxygen or glucose availability diminishes brain function. In addition to stored energy, the brain is highly dependent on the glucose supply from the blood, but glucose metabolism in AD is dramatically decreased. This is likely due in part to enzymes involved in glycolysis, the tricarboxylic acid cycle, and ATP biosynthesis [40]. Thus, glucose content may be a reflection of neuronal function, and the ratio of glucose utilization can be used to observe changes of brain activity in various neurodegenerative diseases.

As an important participant in brain compensation for excessive glucose utilization, brain glycogen metabolism functions to guarantee brain energy and neurotransmitter metabolism. In human and rodent studies, DM affected glycogen content in various brain regions [41]. Compared with that of healthy people, the glycogen content in the occipital lobe of patients with type I DM (T1D) is significantly reduced [42], as is GSK-3*β*, which is a crucial enzyme in glycogen synthesis [23]. Moreover, DM can promote glucose transportation by increasing the permeability of the blood-brain barrier (BBB). In some brain regions, insulin is sensitive to glucose transport proteins (GLUT), such as GLUT4 and GLUT8 [43, 44], and phosphorylation events initiated by insulin signaling are able to regulate GLUT4 transporters [45]. In diabetic animal models, glycolytic ability and acetyl-CoA activity were reduced [41, 46], which contributed to mitochondrial dysfunction by decreasing ATP production and promoting reactive oxygen species (ROS)/reactive nitrogen species (RNS) formation (Figure 1). Apolipoprotein E (APOE) is a multifunctional protein with central roles in lipid metabolism, neurobiology, and neurodegenerative diseases. Neuropathological data and clinical trials [47] have shown that the correlation between DM and AD is particularly strong in individuals carrying the APOE4 allele. Individuals with the APOE4 allele also have lower glucose metabolism in the posterior cingulate, precuneus, and/or lateral parietal regions [48]. Accordingly, diabetic patients with anomalous glucose content and metabolic pathways in the brain have an increased risk of AD.

A variety of preclinical models have shown that energy metabolism is closely related to the pathological development of AD. DM can cause glucose transport disorders and metabolic abnormalities in the body, which can in turn lead to cognitive dysfunction. Therefore, therapeutic strategies to reduce the incidence of AD in diabetic patients should focus on controlling glycemic levels or restoring glucose metabolism. This may also be beneficial in preventive measures designed to delay and slow AD onset and progression.

## 4. Mitochondrial Dysfunction and Oxidative Stress Are Relevant to the Occurrence and Development of AD in Diabetic Patients

Mitochondria have important physiological functions in the body, including oxidative respiration, energy metabolism, free radical production, and apoptosis [49, 50]. Many studies have shown that mitochondria play an essential role in delaying aging and preventing neurodegenerative diseases [51]. However, due to the abundant energy demand of the brain, it is much more vulnerable to mitochondrial dysfunction [52]. Many studies have further shown that mitochondrial dysfunction is indispensable in the pathogenesis of various diseases such as DM and neurodegenerative disorders [51, 53]. Moreover, oxidative phosphorylation creates ROS as a by-product, which may damage different types of molecules and further increase oxidative stress. Oxidative stress is closely associated with age-related diseases [52], and therefore, mitochondria can be both dangerous and beneficial.

Dysfunctional mitochondria are less efficient in producing ATP, but more efficient in generating ROS, which could represent a primary cause of the oxidative imbalance observed in AD [54, 55]. It has been demonstrated that the imbalance of mitochondrial division and fusion may give rise to excessive ROS production, dissipation of membrane potential, deficiency in cellular respiratory function, and decreased ATP production [56]. A large amount of ROS will result in a series of common AD pathological alterations, such as oxidative damage of proteins, carbohydrates, and lipids. Nishikawa et al. have shown that increased mitochondrial oxidative stress and ROS production can occur under hyperglycemic conditions [57]. Dysregulated mitochondria can contribute to calcium dyshomeostasis and cause apoptosis and memory impairment [56, 58, 59]. In addition, the accumulation of mtDNA mutations is one reason for electron transport chain abnormalities, which may affect ATP production, destroy brain function, and facilitate the AD occurrence [56, 58, 59]. These mtDNA mutations have been detected in type II diabetes (T2D) patients, while mitochondrial genes are often missing in pancreatic *β* cells in T2D animal models [60]. Proteins such as peroxisome proliferator-activated receptor gamma coactivator 1*α* (PGC-1*α*), nuclear respiratory factor 1 (NRF1), nuclear respiratory factor 2 (NRF2), and mitochondrial transcription factor A (TFAM) are involved in mitochondrial biosynthesis and are also strongly related to neurodegenerative disorders [61]. With AD initiation, the expression levels of these genes were downregulated and mitochondrial biosynthesis decreased conspicuously [61]. This suggests that disruption and regulation of mitochondrial biosynthesis may be a target for AD treatment. Moreover, PGC-1*α* and PINK1 can decrease the peroxidation of mitochondrial fatty acids and have been found to be significantly reduced in the hippocampus of AD patients and diabetic mice [62]. These data indicate that PGC-1*α* and PINK1 are essential factors in the modulation of mitochondrial function in the brain, which might be an underlying mechanism in diabetic patients' vulnerability to AD.

As a destructive factor, oxidative stress occurs when cellular metabolic activity is greater than antioxidant capacity or when the amount of free radicals (including ROS and RNS) production/accumulation is too large to be removed. The levels of ROS and RNS were obviously elevated in patients of DM and AD [53, 63, 64], indicating that oxidative stress may be a connection between both diseases. Oxidized proteins/lipids are increased in the brain of AD patients, and there is also evidence that oxidative damage can occur in the early stages of patients with moderate cognitive impairment [58, 65, 66]. Impairment of the respiratory chain, which is associated with oxidative stress and malfunctioning mitochondria, is a typical characteristic of streptozotocin- (STZ-) treated rat brains [67]. In line with this, respiration/oxygen consumption is decreased in the brain mitochondria of male STZ diabetic rats [68]. In diabetic rats induced by STZ, thiobarbituric acid reactive species (TBARS) levels were significantly elevated in the hippocampus, while the level of superoxide dismutase (SOD) was markedly downregulated. This was also accompanied by a reduction in mtDNA content and oxidative respiratory chain proteins [54]. Ceretta et al. also found that diabetic rats had an increased level of superoxide, protein oxidation, and TBARS production in some brain regions, while SOD activity was decreased in the striatum and amygdala [54]. Similarly, the activity of superoxide dismutase, catalase, or glutathione peroxidase is often decreased in diabetic brains [69–72]. The resulting excessive oxidative stress may trigger the discharge of cytochrome C and activate the prodeath apoptotic cascade, which in turn promotes mitochondrial dysregulation [73]. Collectively, these data indicate that oxidative stress arises in the brain and that oxidation products are significantly increased in diabetes models.

Taken together, the above results indicate that the mitochondria in diabetic patients and models represent an imbalance between oxidation and antioxidant capacity. This further exacerbates oxidative stress, disrupts mitochondrial dynamics (fission and fusion) and biological functions, and causes mtDNA mutations. All of these events can result in an insufficient energy supply and reduced antioxidant enzyme activities in the brain area, which eventually increases the risk of cognitive dysfunction and memory defects. In general, DM can aggravate mitochondrial dysfunction and oxidative stress in memory- and cognition-related brain regions, which might be the common underlying mechanism to these diseases.

## 5. Abnormal Lipid Metabolism: A Key Feature of Neurodegeneration That Is Exacerbated by DM

Lipids are abundant in the brain, particularly glycerophospholipids, sphingolipids, and cholesterol. It has been demonstrated that lipid oxidation products are at high levels in tissues derived from aged mice [74, 75]. In one study of postmortem AD brains, “adipose inclusions” or “lipid granules” could be found [76]. Physiological and epidemiological investigations have also confirmed that cholesterol metabolism, inflammation, and innate immunity are closely associated with AD [77]. In neurons and other cell types, oligomeric A*β* peptides can alter cellular cholesterol metabolism [78]. This is significant as obesity and dyslipidemia are the main risk factors for T2D, accompanied by increased central adiposity, elevated triglycerides and low-density lipoprotein cholesterol (LDL-C), and reduced high-density lipoprotein cholesterol (HDL-C) [79]. Thus, abnormal lipid metabolism is an overlapping feature of both DM and AD.

Lipids, especially cholesterol, have been discussed at length in the context of neurodegenerative diseases. Disturbance of cholesterol metabolism in the brain is correlated with aging and neurodegenerative diseases such as AD [80]. Cholesterol in the brain is essential for synapse and dendrite formation, as well as axonal guidance [81–83]. However, the BBB can effectively prevent the exchange of substances such as peripheral cholesterol between brain tissue and plasma lipoproteins [84]. Individuals with diabetes often have hyperlipidemia, which can destroy the integrity of the BBB and increase its permeability. As peripheral cholesterol enters into the brain, cholesterol metabolism disorders occur [83]. In addition, hypercholesterolemia can promote A*β* pathology in the brains of many AD patients and further cause oxidative stress, giving rise to mitochondrial dysfunction and structural damage via lipid peroxidation [85–89]. Increased inflammation has been found in the brains of diabetic mice fed a high-fat diet and may be associated with upregulation of A*β* and phosphorylated tau (p-tau) [85]. In a study performed by Vandal et al., high-fat diets led to glucose intolerance, increased soluble A*β*, and memory impairment in 3xTg-AD mice. Strikingly, the authors also found that a single insulin injection reversed the deleterious effects of high-fat diets on memory and soluble A*β* levels, partly through changes in A*β* production and clearance. In addition, the APOE4 allele has been shown to increase the risk of AD in diabetic patients by selectively binding to the A*β* peptide and modulating its aggregation and clearance [90–93]. As such, the results from preclinical assessments and experimental research of AD and DM models support a crucial role for lipid metabolism in the pathogenesis of both diseases.

In addition to cholesterol, fatty acids have been intensively studied in DM and other metabolic diseases, including neurodegenerative diseases. The plasma level of free fatty acids in STZ-induced diabetic rats was significantly higher than that in the control group [89], which is consistent with findings of other diabetic models [94, 95]. However, increased free fatty acids can impair the function of the central nervous system [88]. In addition, AD patients with different levels of memory loss also showed different degrees of metabolic disorders of fatty acids [96]. Consequently, we can conclude that lipid metabolism is a shared link between DM and AD, as well as provides a plausible molecular basis for the pathogenesis, diagnosis, and treatment of diabetic patients who are prone to dementia.

## 6. Protein Changes in AD and DM

Protein oxidative damage can at least partially explain a wide range of age-related diseases, including metabolic disorders such as DM and obesity, cardiovascular complications such as atherosclerosis, and neurodegenerative disorders such as AD [97]. As a global disease, DM not only is characterized by elevated blood glucose and blood lipid levels but also displays a protein metabolic disorder, which is primarily caused by the absolute or relative deficiency in insulin secretion [98]. Insulin can promote protein synthesis and inhibit protein decomposition [99], and increased insulin and insulin-like growth factor 1 (IGF1) levels in the serum can improve the protein metabolism (Figure 1). Compared with that in a normal group, protein catabolism in diabetic models was significantly ameliorated, while protein synthesis and the mRNA expression of the IR, IGF1, and IGF1 receptor (IGF1R) were markedly decreased [100]. In addition, the branched-chain amino acids (BCAAs), leucine, isoleucine, and their related metabolites were positively associated with insulin resistance.

Protein accumulation in an aggregated form is a common feature of neurodegenerative disorders. These proteins also tend to aggregate in compartments that differ from the compartments where they are localized under physiological conditions. Over the last decade, many studies have demonstrated that the unfolded protein response (UPR) markers such as binding immunoglobulin protein (BIP), phosphorylated- (p-) protein kinase double-stranded RNA-dependent (PKR)-like the endoplasmic reticulum (ER) kinase (PERK), p-inositol-requiring enzyme (IRE), and eukaryotic initiation factor 2*α* (p-eIF2*α*) were elevated in the most severely affected tissues of AD patients [101–103]. The most widely studied arm of the UPR response is the PERK-eIF2*α* axis. This axis can facilitate A*β* production and take part in the regulation of synaptic plasticity [104]. UPR and ER stress markers such as nuclear factor-*κ*B (NF-*κ*B) target genes, C/EBP homologous protein (CHOP), and immunoglobulin heavy chain (BIP) in inflamed islets of both diabetic mice and patients with autoimmune diabetes were increased [105, 106]. In addition, elevated UPR or ER stress markers are also correlated with tau pathology [107]. In addition to amyloid plaques, neurofibrillary tangles composed of the microtubule-associated protein tau are another hallmark of AD. It has been shown that A*β* is upstream of tau in the AD pathological cascade [108–111]. In AD, tau is massively phosphorylated, particularly at serine and threonine residues. Generally, hyperphosphorylated tau in the axon detaches from the microtubules and passes through the axon initial segment, which normally acts as a diffusion barrier for physiologically phosphorylated tau, before accumulating in neuronal cell body dendrites. This process is partly mediated by A*β* [112–114]; however, recently, it was shown that A*β* could directly trigger endogenous tau overexpression via protein translation and activation of the Fyn/ERK/S6 signaling pathways in the somatodendritic domain [115]. Recent evidence also suggests that AD-related proteins such as A*β*, islet amyloid polypeptide (IAPP), or tau could promote diabetic phenotypes and further exacerbate neurodegeneration [20].

IAPP or amylin is a key feature of T2D pancreatic pathology [20, 116]. This hormone is secreted with insulin, and its regulation begins with food intake [116]. It has demonstrated that IAPP plays a role in the neurodegenerative process of AD [117, 118], and similar findings in the brains of diabetic patients with AD were observed by Fawver et al. [119]. Colocalization of IAPP and A*β* in the brain has been observed, and IAPP can alter microvasculature and tissue structures in patients with AD [118, 119]. Thus, IAPP provides further data supporting a connection between AD and T2D. Although there are notable protein changes in both AD and DM, whether these changes directly contribute to the prevalence of AD in diabetic patients requires further exploration.

## 7. Metabolism and A*β* Deposition

The common pathological characteristics in DM and AD are the generation of amyloid peptides (APP) and aggregation of abnormal proteins such as elevated serine phosphorylation of IRS-1 levels and activated JNK [33]. Extracellular amyloid plaques consist of insoluble aggregates of A*β*, which is one initiator of AD. Growing evidence supports the concept that a series of changes caused by DM can increase the risk for A*β* pathology in many AD cases [120–122]. Due to its positive effects on APP-related gene expression and *γ*/*β* secretase, oxidative stress can promote the pathology of A*β* [89, 123–125]. In cultured cells, when the concentration of cholesterol ester is increased, there is a proportional elevation in APP [126]. It has been shown that A*β* deposition can be seen in mitochondria and, correspondingly, that A*β* deposition can lead to dysfunctional mitochondria such as mitochondrial swelling, mPTPs, and excessive ROS production [127–134]. Therefore, A*β* has a destructive influence on mitochondrial structure and function. Furthermore, increased levels of A*β* may be dependent on mitochondrial ROS production, and antioxidant compounds can impede mitochondrial dysfunction and reduce A*β* generation [73]. Several mechanisms have been proposed to support the idea that insulin signaling dysfunction may lead to A*β* pathogenesis, which can further impact insulin signaling (Figure 1). This suggests that a self-perpetuating cycle may become established, further exacerbating neurodegeneration [135]. Insulin-degrading enzyme (IDE), a major enzyme responsible for insulin degradation, can also degrade other targets such as glucagon, atrial natriuretic peptide, and the A*β* peptide and regulate proteasomal degradation and other cellular functions [136, 137]. It has been demonstrated that IAPP can interact with A*β* and contribute to A*β* aggregation [138] and conformation [20, 119, 139]. Taken together, a positive feed-forward mechanism may exist between metabolic abnormalities and A*β* pathology, which may be explained by the overlapping crosstalk between DM and AD.

## 8. Conclusions and Perspectives

**Figure 2:**
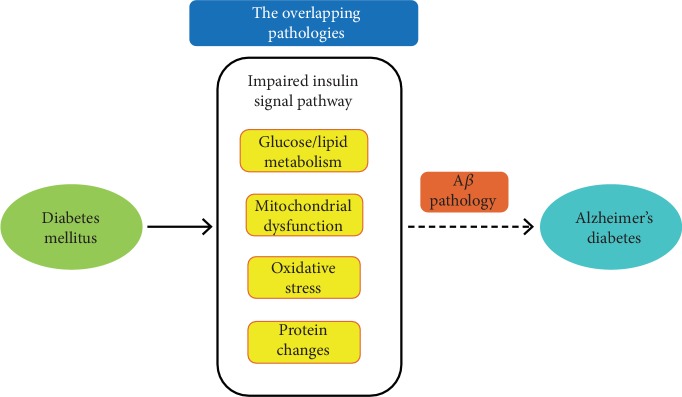
The overlapping pathology between diabetes mellitus (DM) and Alzheimer's disease (AD). Due to many shared characteristics with impaired insulin signaling pathways, AD may be a metabolic disease. Metabolic changes including glucose/lipid metabolism, mitochondrial dysfunction, oxidative stress, and protein changes resulting from DM are associated with an impaired insulin signaling pathway. These metabolic factors could increase the prevalence of AD in diabetic patients, mainly by promoting A*β* pathology.

Multiple epidemiological studies have shown an increased risk of AD in many diabetic patients. As there are many shared characteristics between impaired insulin signaling and AD, it is possible that AD is a metabolic disease. Abnormal glucose and lipid metabolism, mitochondrial dysfunction, oxidative stress, and protein changes resulting from DM are associated with impaired insulin signaling pathways. These metabolic factors can increase the prevalence of AD, mostly by promoting A*β* pathology (Figure 2). Thus, DM is a risk factor for AD that is likely driven by metabolic alterations that exacerbate brain bioenergetic dysregulation. But whether these metabolic factors caused by DM are direct causes or confounders of AD deserves further discussion. Bioinformatics analysis such as Mendelian randomization or genome-wide analysis may help explain the causal relationship between DM and AD. In addition, despite these commonalities, not all DM patients develop AD and not all AD patients are diabetic. In this review, we have explored the overlapping metabolic pathways of DM and AD, which will provide references and suggestions for the underlying mechanisms of diabetic patients with AD. In other words, diabetic patients are more prone to dementia when they have significant metabolic changes such as increased ROS production, abnormal lipid metabolism, and glucose metabolism disorders. In the future, it will be indispensable to clarify whether cognitive decline in diabetic patients can be rescued by adequate metabolic regulation. In addition, it is worth pursuing a comprehensive and integrated analysis of the molecular mechanisms controlling metabolic processes in developing and mature neurons, in order to develop novel therapeutic approaches tailored to the nervous systems of aged people. Futhermore, the crosstalk between these distinct but interdependent biological processes is constantly tuned by shared effectors and regulators. In conclusion, this review discusses the overlapping pathologies between DM and AD from the perspective of metabolism and may accelerate the discovery of new treatments and improve the understanding of DM and AD pathogenesis and progression. Finally, this review may provide theoretical support and new targets for the prevention and treatment of AD in diabetic patients.

## Figures and Tables

**Table 1:** Summary of epidemiological research on DM and AD in recent ten years.

Year	Country	Effective sample size	Case definition		Underlying mechanisms	Reference
			DM	AD/dementia		
2009	Sweden	1248	Being recorded in the inpatient register system, or use of hypoglycemic drugs, or a random blood glucose level ≥ 11.0 mmol/l at baseline (or HbA1c level ≥ 6.4% at second and third follow-up examinations)	Diagnostic and Statistical Manual of Mental Disorders, revised third edition criteria	Glucose dysregulation	[140]
2010	Japan	135	Glucose tolerance test and diabetes-related factors	The Consortium to Establish a Registry for Alzheimer's Disease guidelines and the Braak stage	Insulin resistance	[141]
2011	America	29	American Diabetes Association glycemic criteria for pre-diabetes	Petersen criteria for mild cognitive impairment	Insulin resistance	[142]
2011	China	25393	At least 2 records of DM within one year during 2000–2007 or who had used either sulfonylureas or metformin as oral antidiabetic medication for more than three months	At least 2 records of a diagnosis of dementia within any 1 year during 2000–2007	Oral antidiabetic drugs could decrease the incidence of dementia in T2DM patients	[143]
2013	Japan	175	The Expert Committee on the Diagnosis and Classification of Diabetes Mellitus	The Diagnostic and Statistical Manual of Mental Disorders, revised 3rd edition	DM-related metabolic abnormalities	[4]
2017	Israel	363	The American Diabetes Association classification	The Diagnostic and Statistical Manual of Mental Disorders, fourth edition criteria	Insulin resistance	[144]
2019	Mexican American	69	Self-reported, HbA1c > 6%	Neuropsychological tests	Cell-free mitochondrial DNA	[145]

## References

[1] McNay E. C., Ong C. T., McCrimmon R. J., Cresswell J., Bogan J. S., Sherwin R. S. (2010). Hippocampal memory processes are modulated by insulin and high-fat-induced insulin resistance. *Neurobiology of Learning and Memory*.

[2] Chornenkyy Y., Wang W. X., Wei A., Nelson P. T. (2019). Alzheimer's disease and type 2 diabetes mellitus are distinct diseases with potential overlapping metabolic dysfunction upstream of observed cognitive decline. *Brain Pathology*.

[3] Morris J. K., Vidoni E. D., Honea R. A., Burns J. M. (2014). Impaired glycemia increases disease progression in mild cognitive impairment. *Neurobiology of Aging*.

[4] Fukazawa R., Hanyu H., Sato T. (2013). Subgroups of Alzheimer's disease associated with diabetes mellitus based on brain imaging. *Dementia and Geriatric Cognitive Disorders*.

[5] Roberts R. O., Knopman D. S., Geda Y. E. (2014). Association of diabetes with amnestic and nonamnestic mild cognitive impairment. *Alzheimers Dement*.

[6] Craft S. (2012). Insulin resistance and AD--extending the translational path. *Nature Reviews. Neurology*.

[7] de Matos A. M., de Macedo M. P., Rauter A. P. (2018). Bridging type 2 diabetes and Alzheimer's disease: assembling the puzzle pieces in the quest for the molecules with therapeutic and preventive potential. *Medicinal Research Reviews*.

[8] Takeda S., Sato N., Rakugi H., Morishita R. (2011). Molecular mechanisms linking diabetes mellitus and Alzheimer disease: beta-amyloid peptide, insulin signaling, and neuronal function. *Molecular BioSystems*.

[9] Ascher-Svanum H., Chen Y. F., Hake A. (2015). Cognitive and functional decline in patients with mild Alzheimer dementia with or without comorbid diabetes. *Clinical Therapeutics*.

[10] Hebert L. E., Weuve J., Scherr P. A., Evans D. A. (2013). Alzheimer disease in the United States (2010-2050) estimated using the 2010 census. *Neurology*.

[11] Alzheimer's Association (2016). 2016 Alzheimer's disease facts and figures. *Alzheimer's & Dementia*.

[12] Hebert L. E., Scherr P. A., Bienias J. L., Bennett D. A., Evans D. A. (2003). Alzheimer disease in the US population: prevalence estimates using the 2000 census. *Archives of Neurology*.

[13] Aubert L., Pichierri S., Hommet C., Camus V., Berrut G., de Decker L. (2015). Association between comorbidity burden and rapid cognitive decline in individuals with mild to moderate Alzheimer's disease. *Journal of the American Geriatrics Society*.

[14] Magaki S., Yong W. H., Khanlou N., Tung S., Vinters H. V. (2014). Comorbidity in dementia: update of an ongoing autopsy study. *Journal of the American Geriatrics Society*.

[15] Tumminia A., Vinciguerra F., Parisi M., Frittitta L. (2018). Type 2 diabetes mellitus and Alzheimer's disease: role of insulin signalling and therapeutic implications. *International Journal of Molecular Sciences*.

[16] Abbondante S., Baglietto-Vargas D., Rodriguez-Ortiz C. J., Estrada-Hernandez T., Medeiros R., LaFerla F. M. (2014). Genetic ablation of tau mitigates cognitive impairment induced by type 1 diabetes. *The American Journal of Pathology*.

[17] Baglietto-Vargas D., Chen Y., Suh D. (2015). Short-term modern life-like stress exacerbates A*β*-pathology and synapse loss in 3xTg-AD mice. *Journal of Neurochemistry*.

[18] Koike M. A., Garcia F. G., Kitazawa M., Green K. N., Laferla F. M. (2011). Long term changes in phospho-APP and tau aggregation in the 3xTg-AD mice following cerebral ischemia. *Neuroscience Letters*.

[19] Folmes C. D., Terzic A. (2016). Energy metabolism in the acquisition and maintenance of stemness. *Seminars in Cell & Developmental Biology*.

[20] Bharadwaj P., Wijesekara N., Liyanapathirana M. (2017). The link between type 2 diabetes and neurodegeneration: roles for Amyloid-*β*, amylin, and tau proteins. *Journal of Alzheimer's Disease*.

[21] Schubert M., Brazil D. P., Burks D. J. (2003). Insulin receptor substrate-2 deficiency impairs brain growth and promotes tau phosphorylation. *The Journal of Neuroscience*.

[22] Schubert M., Gautam D., Surjo D. (2004). Role for neuronal insulin resistance in neurodegenerative diseases. *Proceedings of the National Academy of Sciences of the United States of America*.

[23] Zhang Y., Huang N. Q., Yan F. (2018). Diabetes mellitus and Alzheimer's disease: GSK-3*β* as a potential link. *Behavioural Brain Research*.

[24] Pang Y., Lin S., Wright C. (2016). Intranasal insulin protects against substantia nigra dopaminergic neuronal loss and alleviates motor deficits induced by 6-OHDA in rats. *Neuroscience*.

[25] De Felice F. G., Vieira M. N., Bomfim T. R. (2009). Protection of synapses against Alzheimer's-linked toxins: insulin signaling prevents the pathogenic binding of Abeta oligomers. *Proceedings of the National Academy of Sciences of the United States of America*.

[26] Cardoso S., Correia S., Santos R. X. (2009). Insulin is a two-edged knife on the brain. *Journal of Alzheimer's Disease*.

[27] Gasparini L., Xu H. (2003). Potential roles of insulin and IGF-1 in Alzheimer's disease. *Trends in Neurosciences*.

[28] Vieira M. N. N., Lima-Filho R. A. S., De Felice F. G. (2018). Connecting Alzheimer's disease to diabetes: underlying mechanisms and potential therapeutic targets. *Neuropharmacology*.

[29] Zilliox L. A., Chadrasekaran K., Kwan J. Y., Russell J. W. (2016). Diabetes and cognitive impairment. *Current Diabetes Reports*.

[30] de la Monte S. M. (2014). Relationships between diabetes and cognitive impairment. *Endocrinology and Metabolism Clinics of North America*.

[31] Blennow K., de Leon M. J., Zetterberg H. (2006). Alzheimer's disease. *The Lancet*.

[32] Takano K., Koarashi K., Kawabe K. (2018). Insulin expression in cultured astrocytes and the decrease by amyloid *β*. *Neurochemistry International*.

[33] Bomfim T. R., Forny-Germano L., Sathler L. B. (2012). An anti-diabetes agent protects the mouse brain from defective insulin signaling caused by Alzheimer's disease–associated A*β* oligomers. *The Journal of Clinical Investigation*.

[34] Talbot K., Wang H. Y., Kazi H. (2012). Demonstrated brain insulin resistance in Alzheimer's disease patients is associated with IGF-1 resistance, IRS-1 dysregulation, and cognitive decline. *The Journal of Clinical Investigation*.

[35] Forny-Germano L., Silva N. M. L. e., Batista A. F. (2014). Alzheimer's disease-like pathology induced by Amyloid-*β* oligomers in nonhuman primates. *The Journal of Neuroscience*.

[36] Xie L., Helmerhorst E., Taddei K., Plewright B., van Bronswijk W., Martins R. (2002). Alzheimer's beta-amyloid peptides compete for insulin binding to the insulin receptor. *The Journal of Neuroscienc*.

[37] De Felice F. G., Velasco P. T., Lambert M. P. (2007). Abeta oligomers induce neuronal oxidative stress through an N-methyl-D-aspartate receptor-dependent mechanism that is blocked by the Alzheimer drug memantine. *The Journal of Biological Chemistry*.

[38] Rai S., Kamat P. K., Nath C., Shukla R. (2013). A study on neuroinflammation and NMDA receptor function in STZ (ICV) induced memory impaired rats. *Journal of Neuroimmunology*.

[39] Kamat P. K., Rai S., Swarnkar S. (2013). Okadaic acid-induced Tau phosphorylation in rat brain: role of NMDA receptor. *Neuroscience*.

[40] Butterfield D. A., Halliwell B. (2019). Oxidative stress, dysfunctional glucose metabolism and Alzheimer disease. *Nature Reviews. Neuroscience*.

[41] Sickmann H. M., Waagepetersen H. S. (2015). Effects of diabetes on brain metabolism--is brain glycogen a significant player?. *Metabolic Brain Disease*.

[42] Oz G., Tesfaye N., Kumar A., Deelchand D. K., Eberly L. E., Seaquist E. R. (2012). Brain glycogen content and metabolism in subjects with type 1 diabetes and hypoglycemia unawareness. *Journal of Cerebral Blood Flow and Metabolism*.

[43] Vannucci S. J., Koehler-Stec E. M., Li K., Reynolds T. H., Clark R., Simpson I. A. (1998). GLUT4 glucose transporter expression in rodent brain: effect of diabetes. *Brain Research*.

[44] Reagan L. P., Rosell D. R., Alves S. E. (2002). GLUT8 glucose transporter is localized to excitatory and inhibitory neurons in the rat hippocampus. *Brain Research*.

[45] Leto D., Saltiel A. R. (2012). Regulation of glucose transport by insulin: traffic control of GLUT4. *Nature Reviews. Molecular Cell Biology*.

[46] Sickmann H. M., Waagepetersen H. S., Schousboe A., Benie A. J., Bouman S. D. (2010). Obesity and type 2 diabetes in rats are associated with altered brain glycogen and amino-acid homeostasis. *Journal of Cerebral Blood Flow and Metabolism*.

[47] Peila R., Rodriguez B. L., Launer L. J. (2002). Type 2 diabetes, APOE gene, and the risk for dementia and related pathologies: the Honolulu-Asia Aging Study. *Diabetes*.

[48] Knopman D. S., Jack C. R., Wiste H. J. (2014). ^18^F-fluorodeoxyglucose positron emission tomography, aging, and apolipoprotein E genotype in cognitively normal persons. *Neurobiology of Aging*.

[49] Filosto M., Scarpelli M., Cotelli M. S. (2011). The role of mitochondria in neurodegenerative diseases. *Journal of Neurology*.

[50] Verdin E., Hirschey M. D., Finley L. W., Haigis M. C. (2010). Sirtuin regulation of mitochondria: energy production, apoptosis, and signaling. *Trends in Biochemical Sciences*.

[51] Lin M. T., Beal M. F. (2006). Mitochondrial dysfunction and oxidative stress in neurodegenerative diseases. *Nature*.

[52] Golpich M., Amini E., Mohamed Z., Azman Ali R., Mohamed Ibrahim N., Ahmadiani A. (2017). Mitochondrial dysfunction and biogenesis in neurodegenerative diseases: pathogenesis and treatment. *CNS Neuroscience & Therapeutics*.

[53] Wijesekara N., Goncalves R. A., De Felice F. G., Fraser P. E. (2018). Impaired peripheral glucose homeostasis and Alzheimer's disease. *Neuropharmacology*.

[54] Wang X., Wang W., Li L., Perry G., Lee H. G., Zhu X. (2014). Oxidative stress and mitochondrial dysfunction in Alzheimer's disease. *Biochimica et Biophysica Acta*.

[55] Romano S., Mitro N., Diviccaro S. (2017). Short-term effects of diabetes on neurosteroidogenesis in the rat hippocampus. *The Journal of Steroid Biochemistry and Molecular Biology*.

[56] Sheng B., Wang X., Su B. (2012). Impaired mitochondrial biogenesis contributes to mitochondrial dysfunction in Alzheimer's disease. *Journal of Neurochemistry*.

[57] Nishikawa T., Edelstein D., Du X. L. (2000). Normalizing mitochondrial superoxide production blocks three pathways of hyperglycaemic damage. *Nature*.

[58] Gibson G. E., Sheu K. F., Blass J. P. (1998). Abnormalities of mitochondrial enzymes in Alzheimer disease. *Journal of Neural Transmission*.

[59] Choi J., Ravipati A., Nimmagadda V., Schubert M., Castellani R. J., Russell J. W. (2014). Potential roles of PINK1 for increased PGC-1*α*-mediated mitochondrial fatty acid oxidation and their associations with Alzheimer disease and diabetes. *Mitochondrion*.

[60] Wei F.-Y., Tomizawa K. (2011). Functional loss of Cdkal1, a novel tRNA modification enzyme, causes the development of type 2 diabetes [Review]. *Endocrine Journal*.

[61] West X. Z., Malinin N. L., Merkulova A. A. (2010). Oxidative stress induces angiogenesis by activating TLR2 with novel endogenous ligands. *Nature*.

[62] Haughey N. J., Bandaru V. V., Bae M., Mattson M. P. (2010). Roles for dysfunctional sphingolipid metabolism in Alzheimer's disease neuropathogenesis. *Biochimica et Biophysica Acta*.

[63] Kowluru R. A., Mishra M. (2015). Oxidative stress, mitochondrial damage and diabetic retinopathy. *Biochimica et Biophysica Acta*.

[64] Nikooyeh B., Neyestani T. R. (2016). Oxidative stress, type 2 diabetes and vitamin D: past, present and future. *Diabetes/Metabolism Research and Reviews*.

[65] Ceretta L. B., Reus G. Z., Abelaira H. M. (2012). Increased oxidative stress and imbalance in antioxidant enzymes in the brains of alloxan-induced diabetic rats. *Experimental Diabetes Research*.

[66] Castellani R., Hirai K., Aliev G. (2002). Role of mitochondrial dysfunction in Alzheimer's disease. *Journal of Neuroscience Research*.

[67] Mastrocola R., Restivo F., Vercellinatto I. (2005). Oxidative and nitrosative stress in brain mitochondria of diabetic rats. *The Journal of Endocrinology*.

[68] Katyare S. S., Patel S. P. (2006). Insulin status differentially affects energy transduction in cerebral mitochondria from male and female rats. *Brain Research Bulletin*.

[69] Kumar J. S., Menon V. P. (1993). Effect of diabetes on levels of lipid peroxides and glycolipids in rat brain. *Metabolism*.

[70] Makar T. K., Rimpel-Lamhaouar K., Abraham D. G., Gokhale V. S., Cooper A. J. (1995). Antioxidant defense systems in the brains of type II diabetic mice. *Journal of Neurochemistry*.

[71] Miranda M., Muriach M., Almansa I. (2007). CR-6 protects glutathione peroxidase activity in experimental diabetes. *Free Radical Biology & Medicine*.

[72] Alvarez-Nolting R., Arnal E., Barcia J. M., Miranda M., Romero F. J. (2012). Protection by DHA of early hippocampal changes in diabetes: possible role of CREB and NF-*κ*B. *Neurochemical Research*.

[73] Baglietto-Vargas D., Shi J., Yaeger D. M., Ager R., LaFerla F. M. (2016). Diabetes and Alzheimer's disease crosstalk. *Neuroscience and Biobehavioral Reviews*.

[74] Foley P. (2010). Lipids in Alzheimer's disease: a century-old story. *Biochimica et Biophysica Acta*.

[75] Liu Q., Zhang J. (2014). Lipid metabolism in Alzheimer's disease. *Neuroscience Bulletin*.

[76] Fester L., Zhou L., Butow A. (2009). Cholesterol-promoted synaptogenesis requires the conversion of cholesterol to estradiol in the hippocampus. *Hippocampus*.

[77] Robinson M., Lee B. Y., Hane F. T. (2017). Recent progress in Alzheimer's disease research, part 2: genetics and epidemiology. *Journal of Alzheimer's Disease*.

[78] Grimm M. O. W., Grimm H. S., Pätzold A. J. (2005). Regulation of cholesterol and sphingomyelin metabolism by amyloid-*β* and presenilin. *Nature Cell Biology*.

[79] Mooradian A. D. (2009). Dyslipidemia in type 2 diabetes mellitus. *Nature Clinical Practice. Endocrinology & Metabolism*.

[80] Goritz C., Mauch D. H., Pfrieger F. W. (2005). Multiple mechanisms mediate cholesterol-induced synaptogenesis in a CNS neuron. *Molecular and Cellular Neurosciences*.

[81] de Chaves E. I., Rusinol A. E., Vance D. E., Campenot R. B., Vance J. E. (1997). Role of lipoproteins in the delivery of lipids to axons during axonal regeneration. *The Journal of Biological Chemistry*.

[82] Xue-Shan Z., Juan P., Qi W. (2016). Imbalanced cholesterol metabolism in Alzheimer's disease. *Clinica Chimica Acta*.

[83] Kandimalla R., Thirumala V., Reddy P. H. (2017). Is Alzheimer's disease a type 3 diabetes? A critical appraisal. *Biochimica et Biophysica Acta - Molecular Basis of Disease*.

[84] Kang S., Kim C. H., Jung H., Kim E., Song H. T., Lee J. E. (2017). Agmatine ameliorates type 2 diabetes induced-Alzheimer's disease-like alterations in high-fat diet-fed mice via reactivation of blunted insulin signalling. *Neuropharmacology*.

[85] Vandal M., White P. J., Tremblay C. (2014). Insulin reverses the high-fat diet-induced increase in brain A and improves memory in an animal model of Alzheimer disease. *Diabetes*.

[86] Lim W. L., Martins I. J., Martins R. N. (2014). The involvement of lipids in Alzheimer's disease. *Journal of Genetics and Genomics*.

[87] Kurek K., Wiesiolek-Kurek P., Piotrowska D. M., Łukaszuk B., Chabowski A., Żendzian-Piotrowska M. (2014). Inhibition of ceramide de novo synthesis with myriocin affects lipid metabolism in the liver of rats with streptozotocin-induced type 1 diabetes. *BioMed Research International*.

[88] Bonen A., Tandon N. N., Glatz J. F., Luiken J. J., Heigenhauser G. J. (2006). The fatty acid transporter FAT/CD36 is upregulated in subcutaneous and visceral adipose tissues in human obesity and type 2 diabetes. *International Journal of Obesity*.

[89] Bertram L., McQueen M. B., Mullin K., Blacker D., Tanzi R. E. (2007). Systematic meta-analyses of Alzheimer disease genetic association studies: the AlzGene database. *Nature Genetics*.

[90] Bu G. (2009). Apolipoprotein E and its receptors in Alzheimer's disease: pathways, pathogenesis and therapy. *Nature Reviews. Neuroscience*.

[91] Ferrucci L., Guralnik J. M., Pahor M. (1997). Apolipoprotein E *ε*2 allele and risk of stroke in the older population. *Stroke*.

[92] Roses A. D. (1996). Apolipoprotein E alleles as risk factors in Alzheimer's disease. *Annual Review of Medicine*.

[93] Liu Q., Zerbinatti C. V., Zhang J. (2007). Amyloid precursor protein regulates brain apolipoprotein E and cholesterol metabolism through lipoprotein receptor LRP1. *Neuron*.

[94] Blachnio-Zabielska A., Zabielski P., Baranowski M., Gorski J. (2010). Effects of streptozotocin-induced diabetes and elevation of plasma FFA on ceramide metabolism in rat skeletal muscle. *Hormone and Metabolic Research*.

[95] Snowden S. G., Ebshiana A. A., Hye A. (2017). Association between fatty acid metabolism in the brain and Alzheimer disease neuropathology and cognitive performance: a nontargeted metabolomic study. *PLoS Med*.

[96] Baraibar M. A., Liu L., Ahmed E. K., Friguet B. (2012). Protein oxidative damage at the crossroads of cellular senescence, aging, and age-related diseases. *Oxidative Medicine and Cellular Longevity*.

[97] Radman M. (2016). Protein damage, radiation sensitivity and aging. *DNA Repair*.

[98] Morita M., Gravel S. P., Chenard V. (2013). mTORC1 controls mitochondrial activity and biogenesis through 4E-BP-dependent translational regulation. *Cell Metabolism*.

[99] Ren T., Zhu Y., Xia X., Ding Y., Guo J., Kan J. (2017). Zanthoxylum alkylamides ameliorate protein metabolism disorder in STZ-induced diabetic rats. *Journal of Molecular Endocrinology*.

[100] Wang T. J., Larson M. G., Vasan R. S. (2011). Metabolite profiles and the risk of developing diabetes. *Nature Medicine*.

[101] Hoozemans J. J., Veerhuis R., Van Haastert E. S. (2005). The unfolded protein response is activated in Alzheimer's disease. *Acta Neuropathologica*.

[102] Hoozemans J. J., van Haastert E. S., Nijholt D. A., Rozemuller A. J. M., Eikelenboom P., Scheper W. (2009). The unfolded protein response is activated in pretangle neurons in Alzheimer's disease hippocampus. *The American Journal of Pathology*.

[103] Unterberger U., Hoftberger R., Gelpi E., Flicker H., Budka H., Voigtländer T. (2006). Endoplasmic reticulum stress features are prominent in Alzheimer disease but not in prion diseases in vivo. *Journal of Neuropathology and Experimental Neurology*.

[104] Koss D. J., Platt B. (2017). Alzheimer's disease pathology and the unfolded protein response: prospective pathways and therapeutic targets. *Behavioural Pharmacology*.

[105] Tersey S. A., Nishiki Y., Templin A. T. (2012). Islet *β*-cell endoplasmic reticulum stress precedes the onset of type 1 diabetes in the nonobese diabetic mouse model. *Diabetes*.

[106] Marhfour I., Lopez X. M., Lefkaditis D. (2012). Expression of endoplasmic reticulum stress markers in the islets of patients with type 1 diabetes. *Diabetologia*.

[107] Nijholt D. A., van Haastert E. S., Rozemuller A. J., Scheper W., Hoozemans J. J. (2012). The unfolded protein response is associated with early tau pathology in the hippocampus of tauopathies. *The Journal of Pathology*.

[108] Frandemiche M. L., De Seranno S., Rush T. (2014). Activity-dependent tau protein translocation to excitatory synapse is disrupted by exposure to amyloid-beta oligomers. *The Journal of Neuroscience*.

[109] Jin M., Shepardson N., Yang T., Chen G., Walsh D., Selkoe D. J. (2011). Soluble amyloid beta-protein dimers isolated from Alzheimer cortex directly induce Tau hyperphosphorylation and neuritic degeneration. *Proceedings of the National Academy of Sciences of the United States of America*.

[110] Pooler A. M., Polydoro M., Maury E. A. (2015). Amyloid accelerates tau propagation and toxicity in a model of early Alzheimer's disease. *Acta Neuropathologica Communications*.

[111] Li X., Kumar Y., Zempel H., Mandelkow E. M., Biernat J., Mandelkow E. (2011). Novel diffusion barrier for axonal retention of Tau in neurons and its failure in neurodegeneration. *The EMBO Journal*.

[112] Sohn P. D., Tracy T. E., Son H. I. (2016). Acetylated tau destabilizes the cytoskeleton in the axon initial segment and is mislocalized to the somatodendritic compartment. *Molecular Neurodegeneration*.

[113] Zempel H., Luedtke J., Kumar Y. (2013). Amyloid-*β* oligomers induce synaptic damage via Tau-dependent microtubule severing by TTLL6 and spastin. *The EMBO Journal*.

[114] Li C., Gotz J. (2017). Somatodendritic accumulation of Tau in Alzheimer's disease is promoted by Fyn-mediated local protein translation. *The EMBO Journal*.

[115] Chiti F., Dobson C. M. (2006). Protein misfolding, functional amyloid, and human disease. *Annual Review of Biochemistry*.

[116] Clark A., Saad M. F., Nezzer T. (1990). Islet amyloid polypeptide in diabetic and non-diabetic Pima Indians. *Diabetologia*.

[117] Oskarsson M. E., Paulsson J. F., Schultz S. W., Ingelsson M., Westermark P., Westermark G. T. (2015). _In vivo_ seeding and cross-seeding of localized amyloidosis: a molecular link between type 2 diabetes and Alzheimer disease. *The American Journal of Pathology*.

[118] Jackson K., Barisone G. A., Diaz E., Jin L. W., DeCarli C., Despa F. (2013). Amylin deposition in the brain: a second amyloid in Alzheimer disease?. *Annals of Neurology*.

[119] Fawver J. N., Ghiwot Y., Koola C. (2014). Islet amyloid polypeptide (IAPP): a second amyloid in Alzheimer's disease. *Current Alzheimer Research*.

[120] de la Monte S. M., Tong M. (2014). Brain metabolic dysfunction at the core of Alzheimer's disease. *Biochemical Pharmacology*.

[121] Devi L., Alldred M. J., Ginsberg S. D., Ohno M. (2012). Mechanisms underlying insulin deficiency-induced acceleration of *β*-amyloidosis in a mouse model of Alzheimer's disease. *PLoS One*.

[122] Kroner Z. (2009). The relationship between Alzheimer's disease and diabetes: type 3 diabetes?. *Alternative Medicine Review*.

[123] Jo D. G., Arumugam T. V., Woo H. N. (2010). Evidence that *γ*-secretase mediates oxidative stress-induced *β*-secretase expression in Alzheimer's disease. *Neurobiology of Aging*.

[124] Oda A., Tamaoka A., Araki W. (2010). Oxidative stress up-regulates presenilin 1 in lipid rafts in neuronal cells. *Journal of Neuroscience Research*.

[125] Tamagno E., Guglielmotto M., Monteleone D., Tabaton M. (2012). Amyloid-*β* production: major link between oxidative stress and BACE1. *Neurotoxicity Research*.

[126] Cabezas-Opazo F. A., Vergara-Pulgar K., Perez M. J., Jara C., Osorio-Fuentealba C., Quintanilla R. A. (2015). Mitochondrial dysfunction contributes to the pathogenesis of Alzheimer’s disease. *Oxidative medicine and cellular longevity*.

[127] Ronnback A., Pavlov P. F., Mansory M. (2016). Mitochondrial dysfunction in a transgenic mouse model expressing human amyloid precursor protein (APP) with the Arctic mutation. *Journal of Neurochemistry*.

[128] Jafari A., Noursadeghi E., Khodagholi F. (2015). Brain mitochondrial ATP-insensitive large conductance Ca^+ 2^-activated K^+^ channel properties are altered in a rat model of amyloid-*β* neurotoxicity. *Experimental Neurology*.

[129] Kaminsky Y. G., Tikhonova L. A., Kosenko E. A. (2015). Critical analysis of Alzheimer rsquo s amyloid-beta nbsp toxicity to mitochondria. *Frontiers in Bioscience*.

[130] Kumar A., Singh A. (2015). A review on mitochondrial restorative mechanism of antioxidants in Alzheimer's disease and other neurological conditions. *Frontiers in Pharmacology*.

[131] Pinho C. M., Teixeira P. F., Glaser E. (2014). Mitochondrial import and degradation of amyloid-*β* peptide. *Biochimica et Biophysica Acta*.

[132] Ye C. Y., Lei Y., Tang X. C., Zhang H. Y. (2015). Donepezil attenuates A*β*-associated mitochondrial dysfunction and reduces mitochondrial A*β* accumulation in vivo and in vitro. *Neuropharmacology*.

[133] Leuner K., Schutt T., Kurz C. (2012). Mitochondrion-derived reactive oxygen species lead to enhanced amyloid beta formation. *Antioxidants & Redox Signaling*.

[134] Picone P., Nuzzo D., Caruana L., Scafidi V., Di Carlo M. (2014). Mitochondrial dysfunction: different routes to Alzheimer’s disease therapy. *Oxidative Medicine and Cellular Longevity*.

[135] Mittal K., Katare D. P. (2016). Shared links between type 2 diabetes mellitus and Alzheimer's disease: a review. *Diabetes and Metabolic Syndrome: Clinical Research and Reviews*.

[136] Tundo G. R., Sbardella D., Ciaccio C. (2017). Multiple functions of insulin-degrading enzyme: a metabolic crosslight?. *Critical Reviews in Biochemistry and Molecular Biology*.

[137] Pivovarova O., Hohn A., Grune T., Pfeiffer A. F., Rudovich N. (2016). Insulin-degrading enzyme: new therapeutic target for diabetes and Alzheimer's disease?. *Annals of Medicine*.

[138] Ono K., Takahashi R., Ikeda T., Mizuguchi M., Hamaguchi T., Yamada M. (2014). Exogenous amyloidogenic proteins function as seeds in amyloid *β*-protein aggregation. *Biochimica et Biophysica Acta*.

[139] Yan L.-M., Velkova A., Kapurniotu A. (2014). Molecular characterization of the hetero-assembly of *β*-amyloid peptide with islet amyloid polypeptide. *Current Pharmaceutical Design*.

[140] Xu W. L., von Strauss E., Qiu C. X., Winblad B., Fratiglioni L. (2009). Uncontrolled diabetes increases the risk of Alzheimer's disease: a population-based cohort study. *Diabetologia*.

[141] Matsuzaki T., Sasaki K., Tanizaki Y. (2010). Insulin resistance is associated with the pathology of Alzheimer disease: the Hisayama study. *Neurology*.

[142] Baker L. D., Cross D. J., Minoshima S., Belongia D., Watson G. S., Craft S. (2011). Insulin resistance and Alzheimer-like reductions in regional cerebral glucose metabolism for cognitively normal adults with prediabetes or early type 2 diabetes. *Archives of Neurology*.

[143] Hsu C.-C., Wahlqvist M. L., Lee M.-S., Tsai H.-N. (2011). Incidence of dementia is increased in type 2 diabetes and reduced by the use of sulfonylureas and metformin. *Journal of Alzheimer's Disease*.

[144] Lutski M., Weinstein G., Goldbourt U., Tanne D. (2017). Insulin resistance and future cognitive performance and cognitive decline in elderly patients with cardiovascular disease. *Journal of Alzheimer's Disease*.

[145] Silzer T., Barber R., Sun J. (2019). Circulating mitochondrial DNA: new indices of type 2 diabetes-related cognitive impairment in Mexican Americans. *PLoS One*.

